# Correction to ‘Cul4A is an oncogene in malignant pleural mesothelioma’

**DOI:** 10.1111/jcmm.71156

**Published:** 2026-04-21

M. S. Hung, J. H. Mao, Z. Xu, C. T. Yang, J. S. Yu, C. Harvard, Y. C. Lin, D. T. Bravo, D. M. Jablons, and L. You, “Cul4A is an oncogene in malignant pleural mesothelioma,” *Journal of Cellular and Molecular Medicine* 15, no. 2 (2011): 350–358. https://doi.org/10.1111/j.1582‐4934.2009.00971.x.

In the originally published version of this article, an error was identified in Figure 2B. Specifically, in the panel T1–T8, the band labelled T6 was inadvertently duplicated from T5 during figure assembly. In addition, the actin bands corresponding to T2 and T3 were inadvertently reversed during figure preparation. These errors occurred during figure assembly and presentation. The corrected version of Figure 1 is provided below. These errors do not affect the interpretation of the data or the overall conclusions of the study.

**FIGURE 1:**
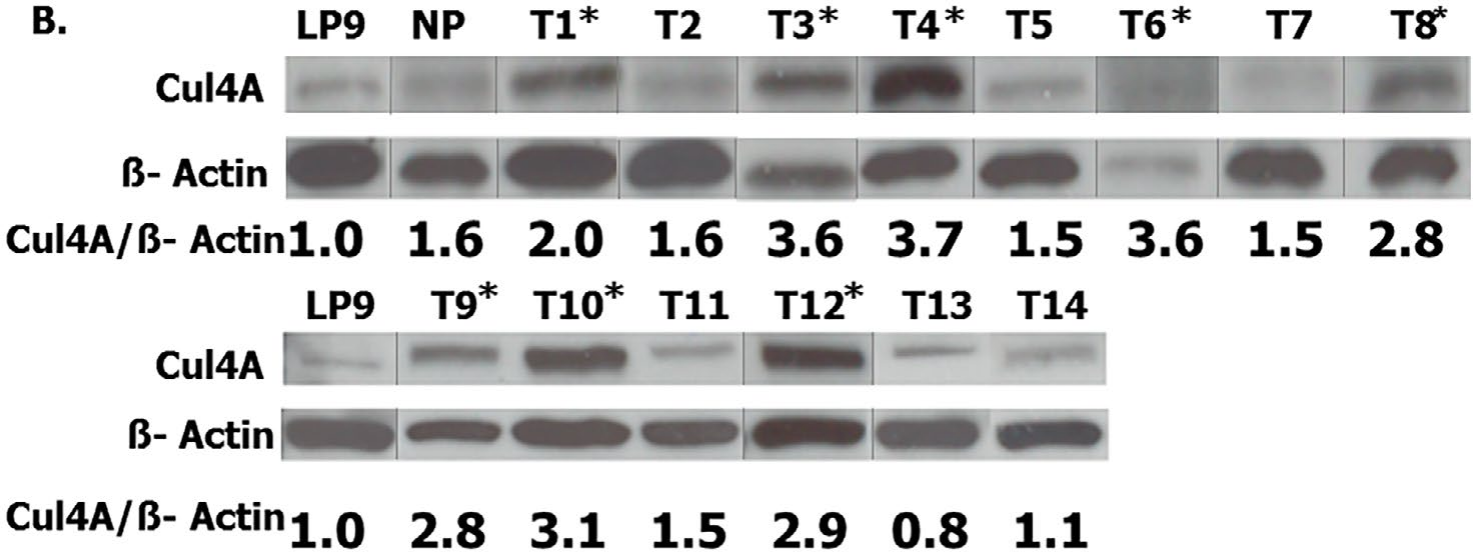
Western blot analysis of Cul4A and β‐actin expression in mesothelioma tumour samples. All lanes were derived from the same original blot; lanes were rearranged during figure preparation for presentation purposes. Splicing boundaries are indicated by lines. LP9 represents a reference/control sample included in both panels. * indicates samples with Cul4A overexpression.

The authors apologize for these errors and for any inconvenience caused.

